# Access to subsidised medicines, cost of medicines and health outcomes: exploring general practitioners' perceptions and experiences

**DOI:** 10.1186/2052-3211-8-S1-O6

**Published:** 2015-10-05

**Authors:** Zaheer-Ud-Din Babar, Abdul Ali, Christine Kim, James Mcintosh, Malaika Samuel Namdas, Erica Lourdes Rodrigues, Komal Vallabh, Anne Rew

**Affiliations:** 1 School of Pharmacy, University of Auckland, Private Mail Bag 92019, Auckland, New Zealand

## Objectives

The general aim of this study was to evaluate general practitioners' perceptions, knowledge and experiences regarding medicines' cost and subsidy in New Zealand.

## Methods

A quantitative cross-sectional study, with postal questionnaires, was conducted to survey 700 New Zealand general practitioners (GPs) registered with the Medical Council of New Zealand. A total of 180 GPs participated, which is 25.7% of the random sample. GPs' perceptions, knowledge and experiences were measured.

## Results

A total of 180 questionnaires were returned and used in the analysis. Of all respondents, only 12% were able to correctly identify the subsidy status of all six medicines. Eighty three percent were of the opinion that the Pharmaceutical Management Agency of New Zealand (PHARMAC) considers its budget as the most important factor when deciding to fund medicines. Only a few participants were able to correctly identify strategies used by PHARMAC. Concerns were raised over the special authority criteria, with 55% agreeing that health outcomes would improve if the criterion was made more relaxed. Fifty-three percent of the GPs agreed that a fully funded medicine improves compliance while 51% agreed or strongly agreed that the increase in medicine co-payment has had a negative effect on patient's access to medicine. Most GPs were neutral to the statements that health outcomes would improve if more new medicines were funded or more medicines in existing drug class were funded.

## Conclusions

Overall, GPs lacked knowledge about what strategies PHARMAC used when deciding to fund medicines. They were deficient in their knowledge about cost of medicines they prescribe. GPs were satisfied with the current range of medicines available and their access to medicines. Most GPs agreed that the increase in medicine co-payment has reduced patients' access to medicines.

